# The meaning of being suicidal - a reflective lifeworld research

**DOI:** 10.1080/17482631.2026.2720834

**Published:** 2026-08-21

**Authors:** Tanja Eriksson, Tabita Sellin, Maria Fogelkvist, Katarina Lindstedt

**Affiliations:** a Department of Psychiatry, Region Örebro County, University Health Care Research Center, Faculty of Medicine and Health, Örebro University, Örebro, SE, Sweden

**Keywords:** Suicide attempt survivors, reflective lifeworld research, being suicidal, suicide attempt, lived experience, phenomenon, essence

## Abstract

**Purpose:**

What drives a person towards death and what it entails to try to kill oneself is not fully understood. This study aimed to examine suicidality as a phenomenon, and to elucidate the essential meaning of being suicidal.

**Methods:**

Applying Reflective Lifeworld Research, the phenomenon of being suicidal was explored through interviews with seven adult suicide attempt survivors (4 men, 3 women; aged 18–54) with various diagnoses. Interviews were analysed using meaning-oriented analysis.

**Results:**

The four essential structures *Fragile vitality versus inherent lethality*, *suicidal isolation*, *incentive of death* and *surrendering to death* constitutes the essence of being suicidal. Being suicidal is a movement from everyday struggle for meaning and value in a perceived meaningless existence, into a fixed suicidal isolation marked by unbearable psychological pain, conviction of worthlessness and exclusion, paralyzing forces and cognitive–existential shutdown. The transition from thought to action is shaped in silent solitude by the incentive of death rather than will or choice. The inner drive towards death appears as a compelling, liberating, inevitable force impossible to resist on one’s own.

**Conclusion:**

This study provides deep understanding about the suicidal mind which may be used in development of assessment instruments and interventions for suicide prevention.

## Introduction

Despite more than half a century of research on suicide, attempted suicide, suicide prevention, and suicide prediction, more than 700,000 people die by suicide each year worldwide (WHO, 2025). Suicide occurs across all regions, under different sociodemographic conditions, by both genders, with or without psychiatric diagnoses and across a wide age range (Franklin et al., 2017; Wasserman, 2016., and Kessler et al., 2020). The suicidal process has been described as highly individual, complicated and fluctuating over time, alternating between latent and acute phases with increasing symptoms prior to a suicide attempt (O´Connor & Kirtley, 2018; Van Orden et al., 2010; Wasserman, 2016). Suicidal ideation (SI) is often viewed as the primary catalyst for suicidal behaviour; however, up to 75% of suicide decedents never reported any such SI (Berman, 2017) and SI may remain undisclosed (Richards et al., 2019). Research has also found that SI may arise only minutes before an attempt (Deisenhammer et al., 2009). It has also been suggested that the period prior to a suicide attempt may be marked by ambivalence between wanting to disclose one’s thoughts and fearing rejection by healthcare providers (Krychiw & Ward-Ciecielski, 2019). Research suggests that the suicidal act can be triggered by intense affects such as hopelessness, rage, abandonment, loneliness, self-hatred and anxiety in combination with a feeling of desperation and an urgent need to escape (Hendin et al., 2007). Other research suggests that an intense feeling of being trapped, in combination with an urgent need for escape where no escape is possible, precede the acute suicidal process (Li et al., 2018; O’Connor & Kirtley, 2018). The period preceding a suicide attempt may be characterised by feelings of emptiness, fear of living, overwhelming emotions, shame and guilt, mental pain, lack of social interaction, self-perception as being a bad person, or a belief that the world would be better without oneself (Bergmans et al., 2017; Shamsaei et al., 2020; Vatne & Nåde, 2012). Suicide attempts may be impulsive (Richards et al., 2019), or may result from long-term suffering, moral considerations and a longing for escape (Vatne & Nåde, 2014), or an inability to cope with one's current life situation (Liang et al., 2020). It may also be experienced as a struggle between wanting to live and wanting to die, or between compassion and safety and the desire for relief from suffering (Bergmans et al., 2017; Vatne & Nåde, 2012).

Although considerable knowledge exists regarding the suicidal process and triggering factors escalating suicidality, we still know comparatively little about the inner drive that pulls a person toward a suicide attempt. Suicidality as a phenomenon—and the transition from SI to suicidal action—is still not fully understood (Klonsky et al., 2021). Learning about individuals’ lived experience of surviving a suicide attempt may provide knowledge and understanding about what propels a person toward a suicide attempt, and what it entails to try to kill oneself. The crucial question is not only who is at risk, but what it is like to reach the point where death comes to appear as a possible or necessary solution. Such knowledge may deepen our understanding of the suicidal person and contribute to more sensitive clinical care as well as more effective suicide prevention.

## Aim

The aim of the study was to examine suicidality as a phenomenon, and to elucidate the essential meaning of being suicidal.

## Material and methods

To be able to capture the meaning of being suicidal, a phenomenological design based on Reflective Lifeworld Research (RLR) was applied (Dahlberg et al., 2008). This method is grounded in Husserl’s assumption that objective knowledge can only be attained through the subject’s experience and perception of the world. The lifeworld (Lebenswelt) is the self-evident, everyday world in which we are always situated—*the taken-for-granted* foundation of experiences, relationships, embodiment, and meaning upon which all science and understanding rest (Husserl, 1970/1936). According to Merleau‑Ponty (2022/1945), the lifeworld is understood as *bodily lived,* meaning that suicidal experiences are accessed not only as cognitive descriptions but as embodied, perceptual realities. The phenomenon, however, is that which presents itself to consciousness as it is experienced (Husserl, 1992/1931). RLR enables the researcher to explore the phenomenon as it appears through the individual’s lived experience (Dahlberg & Dahlberg, 2020).

### Participants

Adults who had survived at least one suicide attempt within the 12 months prior to participation were recruited from general psychiatric outpatient services in central Sweden. Recruitment notices were displayed in waiting rooms and interested patients could contact the research team directly to express interest in participating. In addition, outpatients who met inclusion criteria were informed about the study by healthcare professionals during routine healthcare visits. Those who expressed interest in receiving further information were contacted by the first author (T.E) to assess eligibility for participation.

Patients were not included if they met, at the time of participation, any of following exclusion criteria:

- Ongoing compulsory psychiatric care
- Impaired cognitive ability for any reason
- Being under the influence of alcohol or drugs
- Experiencing severe psychiatric illness such as psychosis

### Data gathering

To explore the lived experience in depth and capture unreflected aspects of the phenomenon, lifeworld interviews were conducted. This interview method aims to allow the phenomenon to reveal itself through the participant’s own expressions. All participants were interviewed once, between September 2021 and March 2022 by the first author (T.E) who had no prior personal, clinical or therapeutic relationship with any of the participants. The interviews lasted between 65 and 122 minutes and resulted in approximately 89,000 transcribed words. Interviews were conducted at locations chosen by the participants and at times convenient for them, and they all choose to be interviewed at the closest hospital or the research centre. All interviews were audio-recorded and transcribed verbatim by an experienced research secretary. The transcripts were then verified by the first author (T.E) to ensure accuracy.

#### Interview structure

In accordance with Dahlberg et al. (2008), the interviews were organised into sequences, and each interview began with an open question designed to direct the participant’s attention toward the phenomenon:

*“You attempted suicide recently/a while ago—can you tell me about it?”*

This was followed by a sequence aimed at guiding the participant’s awareness toward previously unreflected dimensions of the experience, allowing all dimensions of the phenomenon’s meaning to emerge (Dahlberg et al., 2008). Each emerging dimension was explored in the same way until both participant and researcher considered the phenomenon well illuminated and a deep understanding were achieved. Further details of the interview process are presented in Table I.

**Table I.** The sequences of the interview and examples of emerged dimensions of the phenomenon.

Sequence of the interview	Aim of the sequence	Questions and emerged dimensions		
***Opening sequence***	*Question directed towards the phenomenon*	*You attempted suicide recently/a….ago, can you tell me about that?*		
***Directing Sequences***	*Question directing the participant to approach unreflected meaning dimensions of the phenomenon.*	*what did you feel when…*		
		*what did you think about…*		
		*what happened when…*		
		*tell me more about…*		
		*You mentioned… can you tell me more about…*		
		*Example of emerged* *dimensions:*		
***Following up questions***	*In what way?*	*Pause from alcohol*	*Guilt*	*Angry*
	*Can you give an example?*	*I was so sad*	*Self-hate*	*Panic*
	*Can you describe…?*	*I cannot handle it any more*	*Destructive relationship*	*The only way out*
	*How did it happen?*			*Determined*
	*Tell me more!*	*I had suicidal thoughts*	*I was feeling so great*	*Work*
	*What did/does it mean to be/do/feel...*	*I didn’t want to be alone*		*Relation*
			*Don’t want to be admitted to a psychiatric hospital*	*Trapped like a mouse*
		*It was hopeless*		
		*I was irritated*		*Insignificant*
		*Mistrust*	*I was so anxious*	*There was nothing left*
		*Relations*	*Stressed*	
				*Darkness*
		*To be understood*	*No way out*	*Dark thoughts*
		*Someone cares for me*	*Desperate*	*Insecure*
		*Death*	*I was completely*	*Value to someone*
		*Dark hole*	*done*	*To mean something to someone*
			*Anger towards*	
		*Dammed thoughts*	*oneself*	*Shame*
			*Worthless*	
		*Unsuccessful*	*No value*	
			*Black hole*	

#### Preunderstanding

The overall purpose of this study was to conduct credible and reliable research. The findings are thus presented with transparency and without distortion. However, all the authors work clinically in psychiatric care and have extensive experience with suicidal patients, thus the research team’s preconceptions and biases were both solid and ever-present. The first author, a doctoral student and clinical specialist nurse in psychiatric care, conducted all interviews. Co-authors (PhDs) contribute long-standing expertise in clinical psychiatry and qualitative research. Consistent with RLR, we aimed to practice bridling as an active, ongoing stance of openness and restraint—acknowledging and gently limiting preunderstanding so that the phenomenon was allowed to manifest itself as it is on its own terms (Dahlberg et al., 2008). Bridling has been maintained in repeated internal triangulation, in which interpretations were continuously compared with the original data and with our assumptions and preunderstanding. While Husserl (1992/1931) emphasised bracketing (epoché), and Heidegger (2013/1927() stressed reflexive awareness of preunderstanding, bridling offered a practicable middle path during data collection and analysis.

### Analysis

#### Phenomenological approach

The analysis was conducted using phenomenological meaning-oriented analysis as described by Dahlberg et al. (2008). This approach aims to uncover and describe the *essence* of a phenomenon—in this case, the lived experience of being suicidal. A phenomenon’s essence is the invariant structure, that which makes it this very phenomenon and not something else. The essential structures and all the nuances and variations of the phenomenon constitute the essence (Dahlberg et al., 2008), and thus the meaning of being suicidal. The process is characterised by the researcher’s openness towards the phenomenon and sensitivity to the participants’ narratives, while actively bridling preconceived notions. To maintain this openness, the entire research team participated in the analysis, acknowledging our preunderstanding and prejudices. All meanings, clusters of meanings, and constituents were discussed and compared with the original narratives. This iterative dialogue ensured that the phenomenon was illuminated based on the participants’ expressions rather than our assumptions, which allowed us to be receptive. Such receptiveness to the phenomenon’s *otherness* enables the phenomenon to reveal itself as it is (Heidegger, 2013/1927), which is a central principle in phenomenological research.

#### Meaning-oriented analysis

The analysis involved a dynamic movement between the whole and the parts, which unfolded in several phases:

**Familiarisation: the whole**

The research team read the transcripts repeatedly to gain an overall understanding of the material without initiating interpretation.

**Identification of meaning units: the parts**

Passages that appeared essential to the phenomenon were highlighted. Initial interpretations of these meaning units were noted.

**Clustering of meanings: the parts**

Patterns of meaning—similarities, differences, unique and nuances—were sought across all participants. Meaning units with similar significance were grouped into clusters. A temporary pattern of meaning emerged.

**Deepening understanding: the parts—a new whole**

The process was deliberately slowed down. The research team revisited and discussed the clusters multiple times, allowing meanings to change, evolve and reveal themselves as they are. Patterns with similar significance revealed the essential structure that constitutes the phenomenon that emerged.

**Emergence of essence**

Through this iterative process, the condensed core of the phenomenon, the essence, that which makes the phenomenon this very phenomenon and not something else emerged through the essential structures of the phenomenon.

#### Ethical considerations

This study is approved by the Swedish Ethical Review Authority (Dn: 2021-04068). The participants were carefully informed about the intended content of the interview and that some questions could contribute to increased suffering. There was preparedness to act in accordance with local guidelines, in case acute suicide risk were to be discovered during the interview. All participants signed a written consent form before interview and could cancel participation at any time without providing a reason (Vetenskapsrådet, 2017; WMA, 2024).

## Results

Seven participants with a variety of psychiatric diagnoses were interviewed. As described in Table II, the participants represent different genders, a wide age range, mixed methods of suicide attempt and a variety of psychiatric diagnoses. Six participants attempted suicide 1-5 months prior to participation, and one participant attempted suicide 12 month prior to participation. Three participants had done 2 or more suicide attempts during the 12-month window prior to participation.

**Table II.** Participants, method of suicide attempt and variety of diagnoses among participants.

Participants	Suicide attempt prior to participation	Participants mix of diagnoses	Method used in recent suicide attempt
Women: 3	1 month	Bipolar disorder	Traffic incident
Men: 4	2 months	Mixed depression and anxiety	Intoxication with medication only
Age span: 18−54	3 months	Delusional disorder	Intoxication and cutting
	5 months	OCD	Cutting
	12 months	ADHD	Intoxication with medications and alcohol
		Recurrent depression	
		No known disorder	

The presentation of the findings begins with the essence of the phenomenon, followed by the four essential structures that constitute this essence: “Fragile vitality versus inherent lethality”, “Suicidal isolation”, “Incentive of death”, and “Surrendering to death”.

### Essence

The essence of the phenomenon *being suicidal* is a relentless existential struggle within a fragile vitality overshadowed by an inherent drive towards death. Life becomes suspended between a desperate pursuit of meaning and belonging and an existence that drains all strength and renders suffering infinite and unchangeable. A perceived loss of purpose in life, combined with a diminished ability to function in everyday life, results in suicidal isolation. In the phase of suicidal isolation, nothing exists but paralyzing darkness, mental pain and loss of an inner essence. Fear that the overwhelming feelings of worthlessness and insignificance will be validated makes seeking support or care seem discouraging. From this viewpoint, the notion of death emerges as the only conceivable and alluring resolution—a force at once feared and desired. The movement culminates in a final surrender, where the fear of continuing to live with psychological pain and self-hatred outweighs the instinct to survive. This dynamic, constituted by the tension between *fragile vitality and inherent lethality*, *suicidal isolation*, *the incentive of death*, and the process of *surrender to death*, shapes the lived structure of the phenomenon.

### Fragile vitality versus inherent lethality

Participants describe how the movement toward death begins with a lifelong scepticism toward life and one’s own worth. They experience life itself as lacking inherent value, and mere existence is not enough to feel worthy of living. They speak of a lifelong striving to find value in life and existence. Value is perceived as something earned through performance according to personal and societal norms, and participants base their sense of worth on the responses of others. Having a function and feeling a sense of belonging in a relationship, a family, or a social context provides a feeling of significance and creates meaning in existence.

*‘Yes, because otherwise my life is worthless, no one is interested in me, no one cares about me, no one has any use for me’.* Participant no. 7

According to the participants, belonging can offer a sense of security and care. Belonging also means that there is someone who cares about one’s existence, and failure is accepted without altering one’s value. Being involved in a context in which someone relies on them can also provide the external drive needed to take responsibility for regularity and routines. It is easier to endure mental illness when there is always someone who can step in, relieve the burden, and take over responsibility for a while. However, belonging also entails a fear of being hurt, of receiving proof of being insignificant, or of not fitting in. It feels so difficult, complicated, and out of reach that belonging is sometimes avoided altogether.

*‘I'm always chasing security. But at the same time, it feels like I... um... avoid it at all costs.’* Participant no. 4

Life and existence are therefore suspended for a few important but fragile relationships or contexts that participants must struggle to establish and maintain. They describe how exhausting it is just to stay alive. Constantly having to perform, manage anxiety, sleep deprivation, fear of intimacy, fear of loneliness, or cope with drastic mood swings drains one’s vitality and energy. There are only a few things in life that offer rest or respite, and the negative often outweighs the positive. Participants express a lifelong questioning of the purpose of living when life has often felt meaningless.

*‘I felt that somewhere, on some fundamental level, I felt that I shouldn't be alive. Like, what the hell is the point of it all, like... plus I struggle every day and feel like I don't want to... live.’* Participant no. 2

Participants who have previously been exposed to others’ suicides or their own near-death experiences describe how this opened the door to death as a conceivable path. Others speak of an inherent unwillingness to be alive, which has also sparked a natural curiosity about its opposite. They have explored what death might mean and found it to be quiet and restful rather than frightening. The possibility of being able to kill oneself makes life bearable.

### Suicidal isolation

Participants describe how they suddenly find themselves in an existence where a meaningful life is unattainable for the foreseeable future. They speak of losing the ability to alleviate their suffering or find comfort.

*‘I could lie there and stare and stare for hours, for several nights... I stared at the wall and couldn't sleep, even though I had medication, but sometimes the brain is so strong that it takes away the possibility of sleep, no matter what pills I take.’* —Participant no. 7

Those who experience abrupt mood shifts explain how these often lead to panic. Participants describe how the shame of no longer functioning as before and struggling with mental illness makes it unthinkable to turn to family or close ones. They describe how this both triggers and sustains the sense of meaninglessness. Sharing their pain with others feels difficult because it means being reminded of their suffering while simultaneously feeling like a burden. Based on their own or others’ experiences, participants perceive seeking care as unimaginable. They distrust the healthcare system’s ability to relieve or cure them. They also fear being rejected by care providers, as this would confirm their perceived worthlessness. Some express a panic fear of coercive care, stating that the fear of being locked up without the possibility of escape or of finding relief is worse than death.

*“Em, ending up in compulsory psychiatric care. That... is a nightmare. **[researcher: You look really frightened when you say that?]** Yes, I couldn't handle that! Oh my God, that would be… no, ugh... **[researcher: That fear is much stronger than?]** The fear of dying. Because then at least it's my decision to die*!*”*– Participant no. 6

Participants find themselves in both physical and existential isolation. The absence of belonging creates a sense of exclusion and abandonment. They feel overwhelmed by past failures in life, which generates a fear of failing again and prevents them from trying anything new. Participants describe being convinced that they have ruined life for themselves and for others around them. They feel worthless, insignificant, and replaceable. Self-reproach grows without distraction or counterevidence, and participants describe deep self-hatred and intense anger toward themselves.

*‘Yes, but it's like hating someone else. Like you hate someone else so much that you just want to stab them. Like someone who has really let you down badly... and ruined your life.’* **[researcher: But it’s towards yourself].”** *yes!”.*—Participant no. 2

Participants explain how self-hatred and the sense of worthlessness trigger intense psychological pain. Their flow of thoughts is described as fragmented, frenetic, and uncontrollable—or almost completely shut down. The psychological pain also manifests physically as severe anxiety with inner restlessness, a crawling sensation in the body, or shortness of breath. Hunger, pain, and cold are barely registered, or not at all. A pervasive darkness fills the participants and isolates them from all other impressions. In this darkness, only negative thoughts, despair, hopelessness, and resignation remain, enveloping their entire existence. The darkness is also described as a black hole in the chest, draining all previous positive experiences while simultaneously depriving them of hope of anything positive ever again.

*‘But it feels like there's something like that [a black hole] in my chest... that's where all my positive feelings have gone and will never resurface... And, as I said, there's no way to get any of it back.’*– Participant no. 4

Participants describe how the darkness penetrates their entire life/world, and the force to live drains away. They express how the darkness and psychological pain become unbearable. This is experienced as a paralyzing force that absorbs attention, energy, and direction. Their experience of time and space changes. Simultaneously, participants feel a loss of their inner essence. They lose contact with rational capacity, and both bodily and emotional resonance diminish.

### Incentive of death

Participants feel trapped in an existence that seems impossible to alleviate, resolve, or change. A sudden instinct to die arises. The movement toward death occurs subconsciously, and participants feel that all actions inevitably lead toward death. Death is perceived as its own irresistible driving force. For those who have previously attempted suicide, the urge to die comes quickly, without warning, and is triggered by minor events. The movement toward death feels automated, easily accessible, and welcome.

*‘When the... the evil side wins against the one who... wants to stay and... when it takes over too much, then it's like losing control, because it has taken over... all the negative has taken over…more than anything else-* *”* Participant no. 5

Participants may experience feeling compelled to die when they find no other way to escape the darkness and psychological pain. This realisation is perceived as shocking and repulsive. The grief of being forced to die is described as heavy and palpable. Having to leave loved ones behind is perceived as both painful and contradictory.

*“I was completely distraught because I didn't want to leave them... and I was distraught and sad and cried and called out for my children. It was really, really awful.”*– Participant no. 1

It can also feel shameful and degrading to give up the struggle for life. On the other hand, participants describe that trying to kill oneself is experienced as a way to regain control when everything else is out of control. Death is enticing as a place where failure or harming others is no longer possible. Participants imagine that there is rest and respite in death and that suffering is absent. Existence ceases, and nothing ever needs to be managed again. Participants also describe how death feels both logical and reasonable. They portray death as the only positive force to grasp, as it is expected to offer more than life. Regardless of whether death feels liberating or coercive, a sense of calm arises when death is accepted. Psychological pain ceases and is replaced by a feeling of release. It can even evoke an ecstatic longing.

*“Dam, I'll finally get to do what I've really wanted to do for a very long time.”*– Participant no.2

### Surrendering to death

Participants describe the process of surrendering to death as a complex psychological struggle in which they deliberate with themselves. They doubt their ability to succeed in killing themselves, as they perceive themselves to have failed at everything they have pursued in life. They attempt to justify their decision to die against moral and religious principles. This inner deliberation continues until participants are convinced that there are no reasons left to live, and death appears as an easy and natural choice.

*‘I feel, um... worthless... I think about all the mistakes I've ever made. I think about all the difficult things I've ever been through. And then I think something like, “You stupid idiot, why didn't you do this earlier?’*-Participant no. 6

Participants describe how it feels unfeasible to find lethal means, but instead using what is within reach. They also explain that it is difficult to reason rationally or calculate what might be effective or lethal. They cling to a method based on previous experience or because it feels familiar. The drive and impulse to die can be so intrusive and powerful that there is no space for thoughts or doubts about something’s effectiveness—whatever is at hand will suffice, and death or survival is left to fate.

*‘No, I've never thought about which tablets do what... but if there are a lot of tablets, I’ll take them all and then see what happens, more or less.’ -* Participant no. 7

When the location of the act is important for safety or security, participants must first overcome external obstacles such as distance, terrain, and weather. The location may also hold significance for being seen by others in the moment of death.

*‘My hope was that he would see me die, so that he could see the beauty in my death. If he saw that, he would understand why I longed for it so much. Yes, that is what I want, that is beautiful, that is nothing to be sad about.’*– Participant no. 3

Participants experience the struggle between the drive to die and the reluctance to harm themselves as powerful and uncontrollable. They describe the attempt to kill oneself as almost overwhelming, that survival instincts protect the body from injury, and participants must actively fight to overcome them. In parallel, an inner battle arises to find the courage and strength, where fear of pain, discomfort, or bodily harm are obstacles that must be surmounted. Participants state that ultimately, it is the fear of continuing to live with the darkness and psychological pain that provides the strength and courage needed to kill oneself. Participants’ hatred and disgust of themselves erase the self-respect that stands in the way of harming themselves. Others describe how the drive is so forceful that no additional courage is required.

*‘I know I sat there thinking, “Do I want this?” 'No!' And then I drink it anyway, and it goes black pretty quickly.’*- Participant no. 1

Some participants describe how they prepare to die in solitude without any risk of interruption. They try to protect loved ones from finding the body or hide themselves out of shame. The fear of being worthless persists until the end, and participants describe cleaning their home and caring for their body meticulously to avoid evoking disgust as dead. Others also recount that it feels safer and less lonely to die near loved ones or with their belongings close. It is important to participants to show that death has been chosen soberly, to free others from guilt or prevent any suspicion of crime through letters or messages. By contacting loved ones, participants can offer farewells and seek forgiveness. At the same time, it can feel entirely impossible to communicate anything to the outside world.

*‘I can't at the moment... how does one write a letter/.../you're stuck in a black hole, you can't do things like that!’*—Participant no. 4

Participants describe how anxiety, darkness, and psychological pain are replaced by a quiet anticipation of closure and rest.

*‘So I felt relieved when I realised, “Okay, the pills are kicking in now,” and it was nice being able to pass away’.*—Participant no. 7

Death is described as beautiful and, by visualising their dead body, a longing for freedom emerges. Waiting for the end is described as relaxing; participants feel finished with life and hope not to survive.

## Discussion

To the best of our knowledge, this is the first study using RLR to explore suicidality as a distinct phenomenon with the aim to reveal the essence of being suicidal. The intention was to explore the phenomenon of being suicidal as such, using a transdiagnostic approach that was not restricted to particular diagnoses, gender, or age groups.

### Interpretation of the results

The suicidal lifeworld revealed itself as unstable and fluctuating—lasting minutes for some and years for others. Across accounts, the movement toward a suicide attempt originated in a long-lasting struggle to maintain personal worth, functioning, belonging, and meaning. In this latent phase, the possibility of future suicide appeared both as a lifeline and an escalating crisis plan. Participants felt trapped in worthlessness and perceived meaninglessness, unable to alter circumstances or restore a sense of worth, which they linked to performance, functioning, and self-worth.

The findings of the present study disclose the lived experience of perceived exclusion from any context. This resembles the previously described concept of *thwarted belongingness*—the perceived conviction of no longer belonging to any social context or group (Van Orden et al., 2010). The findings in our study, however, show that the consequences of a perceived loss of belonging also means inability to prove one’s value to others and thus, the participants felt forced to find value in a perceived meaninglessness without any possibility of changing their situation or restoring a sense of worth; they felt insignificant, replaceable and worthless. These findings highlight previous findings that lack of support, empathy, and belonging can act as a triggering factor for suicide attempts (Shamsaei et al., 2020).

Negative life events and *defeat* (i.e., one central concept in the Integrated Motivational- Volitional (IMV) model) are thought to lead to suicidality (O’Connor & Kirtley, 2018), but the present findings show that it is more complex than isolated events and a feeling of failing at life; the participants express how it is the impact of different events on the individual's perception of self, value and belonging that is more crucial than the event itself. Furthermore, the participants experienced life as fragile and exhausting to maintain, leaving no reserve strength when even small triggers occurred. This aligns with previous research showing that the accumulation of multiple losses reshaped their sense of self and belonging so profoundly that both life and the right to live were questioned, which aligns with research showing that diverse events drain energy and impair functioning regardless of their specific nature (Marcinkevičiūtė et al., 2024).

The altered sense of self and perceived loss of value forced the participants into suicidal isolation, a concept resembling *social withdrawal,* which is one of the core components in Suicide Crisis Syndrome (Galynker, 2023). The present study shows, however that suicidal isolation is not an active choice but a response to that altered sense of self, and that the isolation entangles the individual into real or imagined exclusion from context, but also a sense of being unworthy of human participation altogether. They perceived themselves as excluded from the world while simultaneously protecting their dignity by excluding themselves from interaction. This profound sense of worthlessness further intensified participants’ isolation which reflects the lived experience of *perceived burdensomeness* as highlighted by Van Orden et al. (2010) in the Interpersonal theory of suicide. A crucial aspect that reinforced the suicidal isolation was the participants fear of rejection or stigma and validation of the conviction of worthlessness. Though previous research has found that fear of stigmatisation and rejection might escalate suicidal intent (Krychiw & Ward-Ciesielski, 2019; Sjöberg et al., 2025), the participants in our study also highlighted a strong belief that care could not alleviate suffering and feared involuntary institutionalisation without possibility of escape, eliminated all options for support, resulting in even more entangled isolation. Participants experienced the resulting existential isolation as an unbreachable darkness filled with pain and unchallenged and uninterrupted negative thoughts. This finding aligns with previous research showing that isolation triggers dissociation, where suicidal individuals distance themselves from mental pain while simultaneously being enveloped by it (Marcinkevičiūtė et al., 2024). Relatives similarly report perceiving suicidal individuals as surrounded by an impenetrable darkness that renders them unreachable (Hultsjö et al., 2019; Shamsaei et al., 2020) and loss of self-initiated suicide as a response to that loss rather than a choice (Ovox et al, 2024). The findings in this study corroborate these observations, showing that participants experienced a state of mental isolation—detached from both self and the world—defined solely by paralyzing suffering. In their isolation, participants experienced a continuum of self-contempt and failure that functioned as an unavoidable force drawing them into the darkness of negative thoughts, strong affects and anhedonia—closely aligned with *psychache* (Shneidman, 1993).

While consistent with findings that depressed suicidal individuals experience more intense affects than non-suicidal depressed patients (Conner et al., 2022; Hendin et al., 2007), the present results, however, indicate that these affects emerge in rapid, cascading sequences rather than independently, leaving participants without means to regulate or alleviate them.

The self-hatred was the catalyst for further development of the participants suicidal isolation, aligning with previous research that has identified self-hatred as a catalyst in the suicidal process among men who survived suicide attempt (Mason et al., 2022). However, the findings in our study show that the self-hatred also triggered the engulfing darkness and rendered endurance impossible, echoing the concept of *desperation* known as an intense feeling of mental pain and anguish, together with the conviction that there is no way out (Hendin et al., 2007). This interplay of self-hatred, mental pain and perceived darkness ensnared the participants to unbearable states of suffering that elicited a panic-driven need for immediate escape and relief, corresponding to the concept of *entrapment* (Gilbert & Allan, 1998), acknowledged as a central moderator anchoring a suicidal crisis (Galynker, 2023; O’Connor & Kirtley, 2018). The firm conviction that the suffering would never end, resembling the presuicidal phenomenon of *frantic hopelessness* ( Galynker, 2023), made death obvious and, for some participants, the only distorted positive force they could grasp.

The result from the present study shows that the movement from thought to action appear as both individual and complex, fundamentally shaped by death’s incentives in relation to life. In line with previous work, death appeared more inviting than life (Marcinkevičiūtė et al., 2024), and prior attempts, near-death experiences, and vivid imagery render death more accessible - even automatic (O’Connor and Kirtley, 2018). Thus, death was less feared than life and morally reasonable, becoming a powerful incentive. Participants described an immediate sense of relief at the moment death was accepted, followed by an internal debate, followed by a new relief when they gathered enough courage and moral justification to attempt suicide. This resembles previous findings that capability of suicide or suicide attempt is a complex development that is based on the suicidal crisis itself (Bayliss et al., 2024) but also reflects the inevitable course towards suicide that participants experienced in the present study. Participants felt compelled by a force beyond their control, requiring immense mental struggle to overcome survival instincts, while self-hatred and fear supplied the strength to act. These findings confirm that the fear of continued suffering often outweighs the fear of being unable to die, providing the courage to attempt suicide (Shamsaei et al., 2020), and align with work showing that the capability develops during crises and adapts to fear (e.g., of pain) by selecting less painful methods (Bayliss et al., 2024). Some participants used alcohol to increase lethality; others avoided it to signal clarity; some used it after prior encounters where staff had urged them to “try harder” to die. Despite alcohol’s impact on acute risk (Borges et al., 2017), our findings show that alcohol might be used as a means toward the goal. Participants in our study chose the means to die based on what was available in the home, what felt comfortable, safe and clean, which is consistent with previous research (Clark & Blosnich, 2022). Furthermore, some participants were afraid of surviving the attempt with more injuries and thus chose the method based on that fear. This is in line with previous findings that highlight the fact that the choice of method is based on individual preferences, such as the person’s assessment of the method’s lethality, whether the method is perceived as clean or ‘messy’, and how distressing the process of dying itself is expected to be (Biddle et al., 2018). Although high pain tolerance is believed to be decisive in determining capability of suicide (O’Connor & Kirtley, 2018), the findings in this study suggest that the ability to kill oneself is more complex than that; participants felt unable to access their rational thinking or logical actions—an experience consistent with the presuicidal concept of *loss of cognitive control* in the Suicide Crisis Syndrome (Galynker et al., 2025). However, our findings show that the fear of continuing a life in mental pain and darkness provides the courage necessary to attempt suicide and even outweighs any fears, which is already highlighted in previous research (Shamsaei et al., 2020). Although stronger intent to die by suicide is often associated with a higher risk of attempting suicide (Rogers et al., 2017) and less impulsivity (Deisenhammer et al., 2009), participants perceived intent as a drive toward death rather than a choice not to live. At times, the momentum felt automatic, leaving little space for deliberation; method and lethality could be shaped by mere chance—meaning that strong intent can coexist with impaired control and emotional flooding, and an act may be intentional yet impulsively enacted.

### Methodological reflections

We have employed several methods to increase the trustworthiness of this study. The description of participants, the interview context and the interview structure allows the reader to assess the study’s transferability. Furthermore, the participants provided rich and reflective descriptions of their lived experience, in their own words. However, the analysis and findings are based on a single interview occasion, and participants did not review the transcripts or results. This limitation of the study makes it impossible to fully ensure that our pre-understanding did not influence the analysis or reporting, even though the selected quotations demonstrate that the findings originate from the participants’ experiences. The quotations also illustrate how the interviewer (first author T.E.) allowed participants to explore the unreflected aspects of their experience to achieve a deeper understanding of the phenomenon.

The dependability of this study is attributed to the research team’s adherence to a phenomenological attitude rather than a natural attitude which, in phenomenology, is understood as the naïve and unreflected acceptance of the world as it appears.

Lifeworld interviews in accordance with Dahlberg et al. (2008) enabled exploration of the phenomenon from multiple angles based on what held meaning for each participant. This approach also ensured a phenomenological foundation through continuous awareness of intentionality—that consciousness is always directed toward something, and that experience is immediately given meaning (Husserl, 1992/1931). Such awareness prevents the researcher from understanding too quickly or imposing premature interpretations. In line with Heidegger (2013/1927), all understanding is existential and *situated;* phenomena appear in the lifeworld, and understanding arises through *being-in-the-world*. Thus, as researchers, we briefly entered the participant’s lifeworld to grasp and explore the phenomenon together. Dependability was further strengthened through systematic analytic discussions and documentation of interpretive decisions throughout the research process. Credibility was strengthened by involving the entire research team in the analysis. Through repeated internal triangulation we carefully discussed how the participants’ narratives were illuminated and compared the constituents with the original data. Bridling allowed us to approach the phenomenon with openness and restraint, preventing our pre-understandings from directing the interpretation. Consequently, interpretations that challenged prevailing assumptions were accepted and illustrated, even when they initially felt uncomfortable. This reflexive approach strengthens the study’s confirmability. Although we sought to minimise bias, the study could not have been conducted without the knowledge we brought into the project; nonetheless, bridling helped keep that knowledge from distorting the findings. It is a time-consuming process, yet it has allowed the research team to understand the phenomenon of being suicidal in a new way, which has led to several important insights. It was a challenge to step back during the analysis and let the phenomenon reveal itself as it is, rather than forcing premature conclusions. By applying RLR with meaning-oriented analysis, all variations and perspectives of the phenomenon have been illuminated, which strengthens the authenticity of the study by ensuring that multiple variations of the experience were represented.

## Conclusion

The essence of being suicidal, as indicated by the present study’s findings, is a lived movement from a lifelong struggle for meaning and value in a perceived meaningless existence to suicidal isolation marked by cascading, paralyzing forces and cognitive–existential shutdown. The present study both confirms previously identified strong affects such as *social withdrawal, thwarted belongingness, loss of cognitive control, psych ache, desperation* and *entrapment,* and shows that these occur simultaneously as an overwhelming and paralyzing cascade, leaving the suicidal individual unable to function either mentally or physically. A panic fear of seeking care - together with fears of rejection, stigmatisation, confinement, and perceived inability of care to alleviate suffering- embedded the already manifest feeling of being excluded. Thus, the suicidal isolation was fixed, leaving the suicidal individual inaccessible for medical attention or support from others. The transition from thought to action appeared individual and multifaceted, shaped by the incentive of death rather than by deliberative choice or explicit will. It also reflects that the suicidal drive is experienced as an inner drive rather than expressed in words, or as a result of demanding thoughts. At points, the momentum toward death ran on automatic, with minimal deliberation about method or lethality. This undermines the equation of chaos with low intent: a strong intention to die can be present while the act is carried out by cognitive paralysis or emotional excess, leaving death or survival to mere chance. The strong feeling of being trapped with no possible relief while death was perceived as both an inevitable, compelling and liberating driving force were described as impossible to resist on one´s own. Death was perceived as both more attractive and less frightening than life, thus, the participant's primary instinct was to seek relief in death.

### Clinical implications

The lived experience of being suicidal both confirms and reinforces several well-established concepts. These should therefore be useful and reliable for use in the assessment, treatment and care of the suicidal individual. The obvious difficulty of seeking medical attention or support from one’s surroundings on one’s own in a suicidal crisis shows that this responsibility needs to be shifted from the individual to the healthcare system. Furthermore, this study provides deep insights into the suicidal lifeworld and the complex transformation from SI to suicidal act, which may be used in the development of interventions for early detection of suicide crisis and, importantly, early identification and intervention to hinder suicidal isolation. The findings of this study may contribute to the development of screening instruments that capture the drive towards suicide rather than explicit suicidal intention. Further research is required to identify and develop interventions with the aim to encourage suicidal individuals to seek relief through health care, rather than in death.

## Data Availability

Due to ethical considerations, participant confidentiality, and the conditions approved by the relevant ethical review authority, the data cannot be made publicly available. However, parts of transcribed interviews (in Swedish) and cluster of constituents (in Swedish) that support the findings of this study are available from the corresponding author, TE, upon reasonable request.
